# *Inula* L. Secondary Metabolites against Oxidative Stress-Related Human Diseases

**DOI:** 10.3390/antiox8050122

**Published:** 2019-05-06

**Authors:** Wilson R. Tavares, Ana M. L. Seca

**Affiliations:** 1 Faculty of Sciences and Technology, University of Azores, 9501-801 Ponta Delgada, Portugal; wrt-94@hotmail.com; 2 cE3c—Centre for Ecology, Evolution and Environmental Changes/Azorean Biodiversity Group & University of Azores, Rua Mãe de Deus, 9501-801 Ponta Delgada, Portugal; 3 QOPNA & LAQV-REQUIMTE, University of Aveiro, 3810-193 Aveiro, Portugal

**Keywords:** *Inula*, oxidative stress, ROS, secondary metabolites, inflammation, diabetes, neurologicaldamage, cancer, sesquiterpene lactones

## Abstract

An imbalance in the production of reactive oxygen species in the body can cause an increase of oxidative stress that leads to oxidative damage to cells and tissues, which culminates in the development or aggravation of some chronic diseases, such as inflammation, diabetes mellitus, cancer, cardiovascular disease, and obesity. Secondary metabolites from *Inula* species can play an important role in the prevention and treatment of the oxidative stress-related diseases mentioned above. The databases Scopus, PubMed, and Web of Science and the combining terms *Inula*, antioxidant and secondary metabolites were used in the research for this review. More than 120 articles are reviewed, highlighting the most active compounds with special emphasis on the elucidation of their antioxidative-stress mechanism of action, which increases the knowledge about their potential in the fight against inflammation, cancer, neurodegeneration, and diabetes. Alantolactone is the most polyvalent compound, reporting interesting EC_50_ values for several bioactivities, while 1-*O*-acetylbritannilactone can be pointed out as a promising lead compound for the development of analogues with interesting properties. The *Inula* genus is a good bet as source of structurally diverse compounds with antioxidant activity that can act via different mechanisms to fight several oxidative stress-related human diseases, being useful for development of new drugs.

## 1. Introduction

Oxygen metabolism, which involves mainly redox reactions, is fundamental for human life, but it leads to the production of reactive oxygen species (ROS) and reactive nitrogen species (RNS) [1,2], affecting regulation of several biological processes and cell functions [3]. ROS and RNS include not only radical species such as hydroxyl radical (^●^OH), superoxide radical anion (O_2_^●−^), and nitric oxide radical (^●^NO), having unpaired electrons and exhibiting short biological half-lives, but also labile nonradicals species like singlet oxygen (^1^O_2_), peroxynitrite (ONOO^−^), and hydrogen peroxide (H_2_O_2_), which can also be transformed into some of the radical species mentioned above [4,5]. All these species, due their irreversible and nonselective reactivity, are associated with oxidative-stress related damage [4]. In fact, when cellular production of ROS and RNS overwhelms the antioxidant capacity of cells, it leads to a state of oxidative stress, which in turn can cause oxidative damage to large biomolecules such as proteins, lipids, and deoxyribonucleic acid (DNA) [6]. The consequent degradation of cellular integrity and tissue functions culminates in the development or aggravation of some disorders such as inflammation, ageing, diabetes, cancer, cardiovascular, neurodegenerative disease, and obesity [6,7,8,9].

A recent topic of increasing interest and investigation in the scientific community is the use of plants and their secondary metabolites as therapeutic agents [10,11,12,13]. Plants are an excellent source of compounds with pharmacological potential and/or possessing leading chemical structures in the development of new drugs [10,11,12], and they have always been used effectively as medicine for treatment of human diseases. The *Inula* species (more than 100 species [14]) from the Asteraceae family (also known as Compositae) are widely distributed in Africa, Asia, and Europe and have been reported to possess more than 400 compounds, mainly terpenoids (sesquiterpene lactones and dimers, diterpenes, and triterpenoids) and flavonoids, with many of them exhibiting interesting pharmacological activities [12,13], and are of great scientific and medicinal interest, as evidenced by the two ongoing clinical studies involving herbal preparations containing *Inula* species (ClinicalTrials.gov Identifier: NCT03256708 and NCT02918487). Furthermore, many studies continue to be published showing the potential of *Inula* species in the treatment and prevention of diseases related to oxidative stress, showing traditional medicine applications of plant, in vitro, and in vivo biological activities of *Inula* extracts. In the Kashmir Himalayas, the roots and seeds of *Inula racemosa* Hook. f. are used to treat various health conditions including inflammation and rheumatism [15], while in Pakistan, to treat rheumatism, they use *Inula orientalis* Lam. (syn. *Inula grandiflora* Willd) [16]. The ethanol extract of *Inula helenium* L. exhibits antioxidant and anti-neuroinflammatory activities in lipopolysaccharide (LPS)-stimulated BV-2 microglia cells, suggesting that the extract could act by inhibiting NO production and inducible nitric oxide synthase (iNOS) expression levels through suppression of the expression of interleukin-6 (IL-6) levels [17]. Qun et al. [18] revealed that the hydroethanolic extract of *Inula helenium* presented anti-inflammatory activity in a mouse model, acting by inhibition of tumor necrosis factor-α (TNF-α)-induced activation of nuclear factor kappa-B (NF-κB) and the expression of IL-1, IL-4 and TNF-α, as shown by the test in human keratinocyte HaCat cell line. Another study [19], revealed that ethanol extract from flowers of *Inula japonica* Thunb. inhibited lipid accumulation in 3T3-L1 adipocytes in vitro and reported also that C57BL/6J mice models fed with high-fat diet with 2.5 g of the extract showed a decrease in body fat mass, hepatic lipid accumulation, and body weight gain, while increasing muscle weight.

The taxonomy of some *Inula* species, as in many other genera, has been altered in recent years, and in this review, only the published works involving species whose binominal Latin name is considered by the “The Plant List” database [14] as an *Inula* accepted name are considered. The abovementioned studies are only a few examples of the great interest in *Inula* anti oxidative-stress related disorders research, which led to an increase in the investigation of the metabolites responsible for the activities exhibited, providing support for *Inula*’s use in traditional medicine, as well as establishing the *Inula* genus as a source of antioxidant compounds. This paper intends to provide a critical bibliographic review that demonstrates this, showing a selection of *Inula* compounds with the highest pharmacological potential for the treatment of oxidative-stress related pathological problems as well as to discuss the mechanisms of action involved in their pharmacological action.

## 2. Radical Scavenging Activity of Secondary Metabolites from *Inula* Species Determined Using DPPH and ABTS Methods

**Figure 1:**
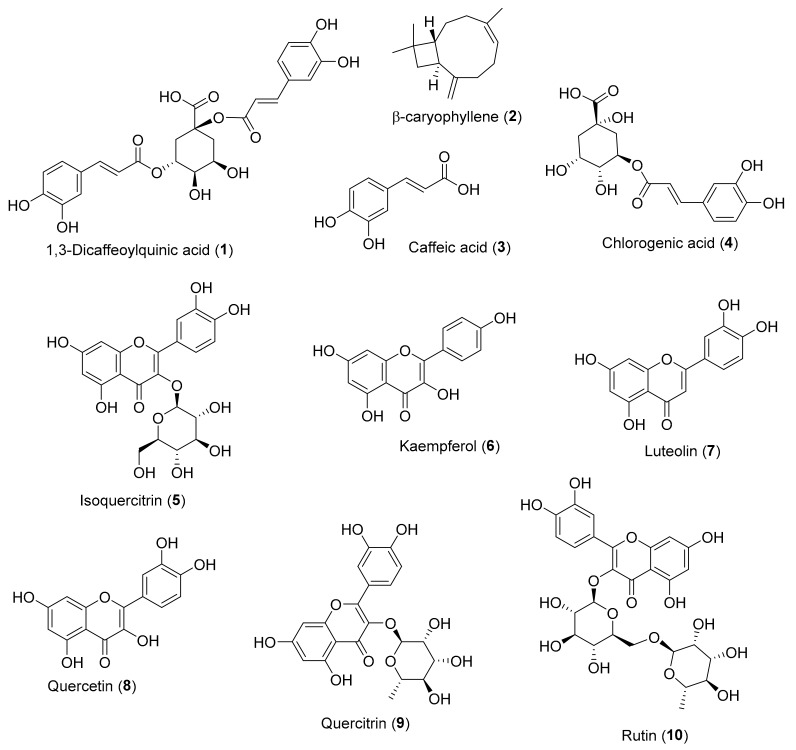
Chemical structure of *Inula* secondary metabolites (**1**–**10**) with DPPH and/or ABTS antioxidant activity.

There are many methods available to allow a first approach for evaluating the antioxidant potential of a compound or extract [20]. Among them, the 1,1-diphenyl-2-picrylhydrazyl (DPPH) and 2,2’-azino-bis(3-ethylbenzothiazoline-6-sulphonic acid) (ABTS) free radical scavenging colorimetric methods are the most popular, since they offer advantages of being rapid, simple, and inexpensive and provide first-hand information on the overall antioxidant capacity of the tested sample [21,22]. However, the two methods are not equivalent: The DPPH scavenging test measures the ability of a compound to neutralize the DPPH radical by a mechanism involving single-electron transfer (SET), while in ABTS assay, the radical neutralization mechanism is mainly hydrogen-atom transfer (HAT), although in some cases, it could also be electron transfer, resulting in a more sensitive method [23,24]. As already mentioned, more than 400 secondary metabolites isolated from *Inula* species are known, and many of them exhibit radical scavenging properties by DPPH and/or ABTS methods. A critical non-exhaustive selection of the most representative *Inula* secondary metabolites, which exhibit an activity identical or superior to that of a reference compound, are presented in Table 1, and the respective chemical structures are shown in Figure 1. In addition, in this selection, we preferentially consider the published works in which the authors present an associated statistical parameter, thus guaranteeing the reliability of the result, and a low associated error (*c.a.* 10% of the mean).

In some assigned cases (see Table 1 note), there was the necessity to convert the EC_50_ values from the original bibliographic source from μg/mL to μM, to allow a comparison of antioxidant activity between the compounds.

According to the DPPH assay values in Table 1, β-caryophyllene (**2**), with an EC_50_ of 1.25 ± 0.06 μM, is by far the most active compound, followed by quercetin (**8**) and quercitrin (**9**), also with interesting EC_50_ values (EC_50_ < 10 μM). It should be noticed that all these compounds showed better EC_50_ values than the reference compound used in their studies, i.e., ascorbic acid or trolox.

As it is possible to see in Table 1, regarding the ABTS assay, a lot fewer published results are available in the literature. Quercetin (**8**) and caffeic acid (**3**) are the compounds with the lowest EC_50_ values, i.e., 6.25 ± 1.09 μM and 8.82 ± 0.33 μM, respectively. Both compounds presented better radical scavenging activity than the reference compound ascorbic acid.

The higher sensitivity of the ABTS method is reflected in lower EC_50_ values when compared to those obtained by the DPPH method for the same compound tested.

It should be emphasized that the results of DPPH and ABTS are somewhat dependent on the used experimental conditions, and therefore, different works may report different DPPH and ABTS EC_50_ values for the same compound (see example: Kaempferol (**6**), Table 1). To mitigate this, it is very important to present the EC_50_ value of an appropriate reference, thus allowing a more reliable comparison of the level of activity in the different publications. Surprisingly, even in recent publications, a significant number of published papers continue to be found that do not meet this requirement. This is a point at which researchers and the peer review process should be more demanding and rigorous, contributing greatly to making the published data more comparable and therefore more useful and of greater impact.

The data in Table 1 show that *Inula* species have relevant compounds with great antioxidant activity, many of them more active than some of the reference compounds, such as ascorbic acid, already used by industry as antioxidants.

Although the antioxidant activity assays by the DPPH and ABTS methods are simple, rapid, and very useful as a first approach, the extrapolation of their results to the antioxidant effect at a cellular level in a biological environment is impossible, and they do not give any information about the cellular mechanisms in which the compounds tested act. This information is very relevant and is obtained using methods and approaches very different from those discussed so far.

## 3. Secondary Metabolites from *Inula* Species against Oxidative-Stress Related Diseases

As noted above, compounds isolated from *Inula* species exhibit a wide range of biological activities against oxidative stress diseases such as inflammation, diabetes, cancer, and neurodegenerative diseases. Thus, much research has been developed to understand how *Inula* compounds act, using models more complex than the model of radical scavenging referred in point 2, and therefore closer to real biological systems. In this section, we present not an exhaustive compilation but rather a critical analysis of the more in-depth studies and the most relevant aspects of the action mechanisms exhibited by the *Inula* compounds that have, as a final consequence, the reduction of the oxidative stress nature inherent to the mentioned diseases.

### 3.1. Inflammation

Since overproduction of ROS leads to cellular and tissue damage, inflammation is intrinsically linked to oxidative stress [42]. Inflammation is a complex defense mechanism that is vital to health since it is the immune system’s response to harmful stimuli, such as damaged cells, toxic compounds, pathogens or irradiation [43]. Cellular and molecular events are triggered in an acute inflammatory response in order to mitigate the impact of an injury or infection, allowing restoration of tissue homeostasis [44]. However, uncontrolled acute inflammation may become chronic, leading to the development of a variety of chronic inflammatory diseases [45]. Intracellular inflammatory signaling pathways include NF-κB, the mitogen-activated protein kinase (MAPK) and Janus kinase/signal transducer and activator of transcription 3 (JAK/STAT3) pathways. All of them are activated by inflammatory stimuli such as TNF-α, interleukin-1β (IL-1β), and IL-6 that interact with the Toll-like receptors (TLR), TNF receptor (TNFR), IL-1 receptor (IL-1R), and IL-6 receptor (IL-6R), mediating inflammation through the production of more inflammatory stimuli [46]. NO is also fundamental in the cellular defense mechanism of inflammation, since NO synthase is induced by pro-inflammatory cytokines; however, it can cause adverse effects such as autoimmune reactions and neurodegenerative syndromes when overproduction of NOs occurs [47]. Cyclooxygenase 2 (COX-2) is a prostaglandin–endoperoxide synthase 2 enzyme that is responsible for generation of prostanoids like prostaglandin E2 (PGE2) that act in the modulation of multiple inflammation and pro-carcinogenic processes [48,49]. The overexpression of COX-2 has been associated with carcinogenesis, resistance to apoptosis, and inflammatory diseases [50,51]. COX-2 expression is controlled by the binding of many trans-factors to the corresponding sites on its promoters, like NF- κB, which in turn, depends on the degradation of IκB proteins by an IκB kinase (IKK) complex [52].

Direct myocardial injury can be caused by inflammatory cytokines response, microcirculation dysfunction, and insufficient energy [53]. The work of Huang et al. [54] clarifies the mechanism by which isoquercitrin (**5**) (Figure 1) attenuates the inflammatory response on LPS-induced cardiac dysfunction on C57BL/6 mice or H9c2 cardiomyoblasts. After LPS stimulation, production of large amounts of TNF-α, monocyte chemoattractant protein 1 (MCP1), and IL6 (all pro-inflammatory cytokines) starts, regulated via the NF-κB signaling pathway, leading to cardiac injury. According to this study, pretreatment with isoquercitrin (**5**) (40 μM) attenuates LPS-induced cardiac dysfunction as well as decreases the levels of TNF-α, IL6, MCP1, and iNOS in vivo and in vitro by blocking the MAPK and NF-κB pathways.

**Figure 2:**
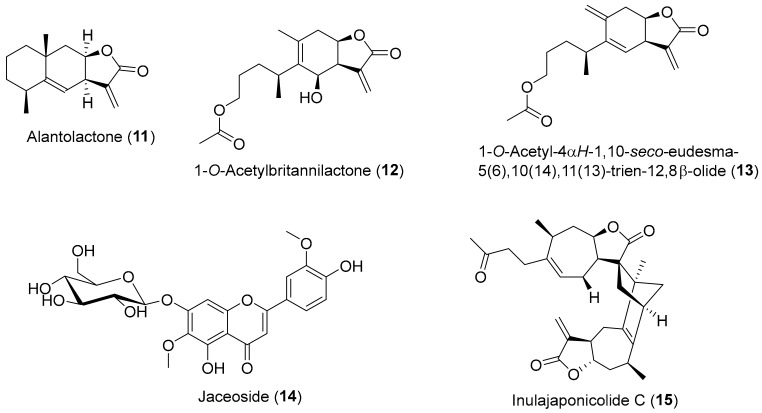
Chemical structure of *Inula* secondary metabolites (**11**, **12**, **14**, **15**) and the semisynthetic derivative (**13**) with reported activity against oxidative-stress inflammatory process.

Alantolactone (**11**) (Figure 2) is a eudesmanolide sesquiterpene lactone with an α-methylene–γ-lactone moiety that is considered the active principle of *Inula helenium* [55]. Alantolactone (**11**) is found in several *Inula* species besides *Inula helenium*, e.g., *Inula japonica*, *Inula racemosa*, *Inula royleana* DC., and *Inula falconeri* Hook.f. [12]. Zhang et al. [56] showed that alantolactone (**11**) inhibits LPS-induced NO production in RAW 264.7 macrophages, presenting an IC_50_ value of 7.39 ± 0.36 μM, being better than the positive control aminoguanidine (IC_50_ = 9.12 ± 0.35 μM). These results are in accordance with the ones presented by Chun et al. [57], where compound **11** at 10 μM inhibited the production of NO, PGE2, and TNF-α, as well as COX-2 and iNOS protein and mRNA transcription in LPS-stimulated RAW 264.7 cells. The same study showed that alantolactone (**11**) disrupted the NF-κB signaling pathway through inhibition of the phosphorylation of inhibitory κB-α (IκB-α) and IKK, as well as the MAPK pathway. A recent study [18] with HaCat cell line revealed that alantolactone (**11**) presented anti-inflammatory activity, since it also could inhibit the expression of IL-1, IL-4, and TNF-α and TNF-α-induced activation of NF-κB, in a dose-dependent manner.

1-*O*-acetylbritannilactone (**12**) (Figure 2) is a 1,10-*seco*-eudesmanolide sesquiterpene that, like compound **11**, has an α-methylene–γ-lactone skeleton, found in *Inula britannica var. chinensis* and *Inula japonica* [12], and that possesses cytotoxic potential [58,59] and anti-inflammatory properties [60,61]. A recent study by Wei et al. [62], found that the 6-deoxy1-*O*-acetylbritannilactone with a methylene at C-14 position, an analogue of 1-*O*-acetylbritannilactone (**12**) labelled as 1-*O*-acetyl-4α*H*-1,10-*seco*-eudesma-5(6),10(14),11(13)-trien-12,8β-olide (**13**) (Figure 2), exhibits an anti-inflammatory effect. In fact, compound **13** decreased NO production and iNOS expression in RAW 264.7 macrophage normal cell line with IC_50_ value of 1.3 μM.

Several compounds from *Inula montana* L. possessed promising anti-inflammatory activity through inhibition of NO production in murine macrophages RAW 264.7 cell line, jaceoside (**14**) (Figure 2) being the compound most active with IC_50_ of 0.34 ± 0.01 μM, being several times better than the positive control drug dexamethasone (IC_50_ of 3.89 ± 0.94 μM) [63].

Several dimeric- and trimeric-sesquiterpenes isolated from *Inula japonica* exhibit anti-inflammatory properties [64]. One of them, the 2,4-linked sesquiterpene lactone dimer named inulajaponicolide C (**15**) (Figure 2), presented the most potent inhibitory effect over NO production in LPS-stimulated RAW 264.7 cells with IC_50_ value of 1.0 ± 0.1 μM, being much better than the indomethacin (IC_50_ = 14.6 ± 0.5 μM) used as positive control.

### 3.2. Diabetes

Diabetes mellitus is characterized by chronic hyperglycemia resulting from flaws in insulin action, insulin secretion, or both [65]. Hyperglycemia induces the increase of ROS production, which in turns causes damages in cells and activation of inflammation processes [66] and triggers apoptosis in the β-cells, worsening the lack of insulin [67]. Thus, acquired insulin resistance and glucose intolerance are associated with chronic inflammation [68,69], the pro-inflammatory cytokine being IL-6 the main link between both processes [70].

A randomized double-blind clinical trial placebo-controlled performed in 30 patients suffering from impaired glucose tolerance showed that the administration of 400 mg of chlorogenic acid (**4**) (Figure 1) three times a day for 12 weeks decreased fasting plasma glucose and increased insulin sensitivity, despite the fact that insulin secretion decreased [71]. The authors suggest that the antidiabetic effect of chlorogenic acid (**4**) could be due to its action on hepatic peroxisome proliferation-activated receptor α (PPARα), which plays a role as a facilitator in clearing lipids from the liver and enhancing insulin sensitivity [72].

The most significant component of the regulating post-prandial insulin secretion mechanism is glucagon-like peptide-1 (GLP-1) that is secreted from cells in the gastrointestinal tract in response to nutrient absorption [73]. GLP-1 is rapidly inactivated in vivo by circulating dipeptidyl peptidase 4 (DPP-IV) [74]. A recent study [75], using colorectal adenocarcinoma NCI-H716 cells as an in vitro model of gastrointestinal cells, showed that isoquercitrin (**5**) is a promising compound to treat type 2 diabetes since it was identified as a DPP-IV inhibitor, with an IC_50_ of 96.8 μM. Furthermore, the levels of GLP-1 increased, suggesting that isoquercitrin (**5**) may also stimulate GLP-1 secretion and bioavailability in a dose-dependent manner. In addition, the same work [75] using in vivo assays with type 2 diabetic Chinese Kunming mice showed that isoquercitrin (**5**) treatment for 8 weeks (80 mg/kg b.w. per day), significantly increased GLP-1 and insulin levels in plasma while lowering the fasting blood glucose levels. These results are in accordance with the ones obtained by Huang et al. [76] that reported hepatoprotective potential of isoquercitrin (**5**) (10 and 30 mg/kg b.w. per day) against type 2 diabetes-induced hepatic injury in rats after 21 days of treatment with significant suppression of DPP-IV mRNA level expression.

Kim et al. [77] demonstrated that alantolactone (**11**) (Figure 2) could increase glucose uptake levels, suggesting it as a great candidate for the treatment of insulin resistance and glucose intolerance. In fact, the 4 h pretreatment of L6 rat myoblast cell line with alantolactone (**11**) (at 0.5 μM), followed by 24 h exposure to IL-6, caused a decrease in the IL-6 induced insulin resistance and allowed the increase of glucose uptake levels to the levels of the control group (without exposure to IL-6). Therefore, alantolactone (**11**) possess antidiabetic potential resulting from its effect against IL-6 induced inflammatory process.

### 3.3. Neurological Damages

Formation and deposition of amyloid beta (Aβ) plaques in the brain in excess, a characteristic of Alzheimer disease (AD), can generate oxidative stress, which triggers inflammatory processes and exacerbates the destruction of hippocampal and neighboring tissues [78]. Therefore, in order to ameliorate or prevent the progression of ROS-mediated neurological damages, antioxidants are considered as promising candidates for therapeutics not only in AD but also in other neurodegenerative diseases like Huntington or Parkinson’s [79,80].

There are indications in the literature that alantolactone (**11**) (Figure 2) exhibits relevant properties to combat oxidative stress, not only in inflammatory processes, as noted above, but also in neurological system. In fact, Seo et al. [81] showed that alantolactone (**11**) at 0.1 to 1 μM has neuroprotective effects on mouse cortical neurons since cell viability was little affected by exposure to Aβ_25–35_ (10 μM), preventing also the shortening of dendrite length, in contrast to what happened in the control group exposed only to Aβ_25–35_ (10 μM). In addition, alantolactone (**11**) treatment decreased acetylcholinesterase (AChE) activity and decreased intracellular ROS production in a dose-dependent manner [81]. However, the authors alert that alantolactone (**11**) at high doses (i.e., >5 μM) could act as prooxidant promoting ROS production. Moreover, the administration of alantolactone (**11**) (1 mg/kg b.w.) reverts scopolamine-induced cognitive impairments in male C57BL/6J and C57BL/6J/Nrf2 knockout mouse, indicating that alantolactone (**11**) improves working memory, probably mediated by activation of the nuclear factor erythroid 2-related factor 2 (Nrf2) signaling pathway [81], a factor that modulates the antioxidant response to an oxidant exposure by a increasing the expression of genes encoding antioxidant enzymes, like the glutathione reductase (GSR), γ-glutamylcysteine ligase (GCL), heme oxygenase-1 (HO-1) and NAD(P)H:quinone oxidoreductase-1 (NQO1) [82,83].

Oxidative stress following traumatic brain injury (TBI) can have devastating effects on brain tissues, since it causes oxidase enzymes activation, mitochondrial functions become impaired, membrane phospholipids are destroyed, and several cellular components, such as DNAs, RNA, carbohydrates, lipids, and proteins, are harmed, which ultimately leads to irreversible damage to neuronal cells and brain tissue [84]. A very recent study [85] reported that treatment of TBI in male Sprague–Dawley rats with alantolactone (**11**) (Figure 2) at 10 and 20 mg/kg b.w. alleviated cerebral edema and improved neurological function via anti-apoptosis, anti-inflammatory, and antioxidative pathways. Furthermore, the same study [85] reported that alantolactone (**11**) significantly suppressed COX-2 expression by inhibiting the activation of the NF-κB pathway, diminishing the levels of glutathione disulphide (GSSG) and malondialdehyde (MDA) (products of lipid peroxidation and an important marker of oxidative damage level [86]) while causing in brain tissues after TBI an increase in the level of glutathione (GSH) and in the activity of superoxide dismutase (SOD), the antioxidant first line defense [87].

**Figure 3:**
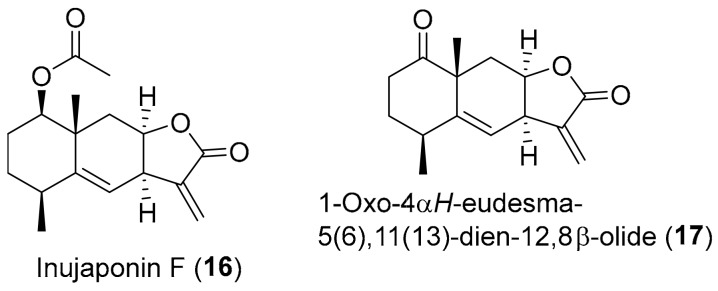
Chemical structure of *Inula* secondary metabolites (**16** and **17**) with reported activity against neurological oxidative-stress damages.

Neurodegenerative diseases like AD are closely related with neuroinflammation [88]. In fact, excessive amount of NO accumulates in the central nervous system (CNS) as a result of inflammatory response over damaged microglia cells, which in turns exacerbates neuroinflammation and aggravates neurodegenerative diseases [89]. Liu et al. [90] isolated various compounds from *Inula japonica*, in an attempt to find potentially useful compounds with NO inhibitory effects for the treatment of neuroinflammation. Inujaponin F (**16**) (Figure 3) and 1-oxo-4αH-eudesma-5(6),11(13)-dien-12,8β-olide (**17**) (Figure 3) presented higher NO inhibitory activity in LPS-induced murine microglial BV-2 cells with IC_50_ values of 1.3 ± 0.1 μM and 1.5 ± 0.2 μM, respectively, higher activity than the one reported by the positive control 2-methyl-2-thiopseudourea sulphate (SMT) that presented an IC_50_ value of 2.9 ± 0.5 μM [90]. This anti-neuroinflammatory effect of compounds **16** and **17**, according to the molecular docking studies, could be due to their ability to interact with residues of the active cavities of iNOS protein, blocking it [90]. The iNOS protein is the most critical component in charge of the amount of NO in inflammatory response [91].

### 3.4. Carcinogenesis

Carcinogenesis is a complex process through which cancer develops, but putting it simple, it basically involves genetic modification of genomic DNA (creation of a mutated cell) followed by growth and division of the aberrant cell with accumulation of additional genetic and epigenetic changes [92]. A recurrent characteristic of cancer progression and resistance to treatment is deregulated redox signaling, which means alteration in redox balance and culminates in elevated levels of ROS [93]. ROS production causes more DNA damage and triggers signaling pathways that activate pro-carcinogenic factors and anti-apoptotic responses, favoring cancer survival and progression [94,95].

Dahham et al. [27] found that β-caryophyllene (**2**) (Figure 1) demonstrated a selective anti-proliferative effect against colon cancer HCT 116 cells (IC_50_ = 19 μM,) and pancreatic cancer PANC-1 cells (IC_50_ = 27 μM), with selectivity index (SI) values from 5.8 to 27.9. It should be pointed out that β-caryophyllene (**2**) presented IC_50_ values not too far from the positive controls 5-fluorouracil (IC_50_ = 12.7 μM) and betulinic acid (IC_50_ = 19.4 μM, SI = 2.7-5). Additionally, β-caryophyllene (**2**) demonstrated apoptotic properties in the HCT 116 cells, by caspase-3 enzyme activation, loss of mitochondrial membrane potential, and DNA fragmentation pathways [27].

An interesting in vitro and in vivo study [96] investigated the effects of alantolactone (**11**) (Figure 2) on several glioblastoma multiforme cells (GBM) (i.e., U87, U251, U118, and SH-SY5Y cell lines) and determined that it suppresses the growth of GBM cells. According to the results, alantolactone (**11**) reduced in a dose- and time-dependent manner the survival rate of the tested cell lines exhibiting the highest cytotoxic activity against U251 cell line (IC_50_ = 16.33 ± 1.93 μM), without displaying cytotoxicity against normal human glial cell line, SVG, at concentrations below 25 μM. Furthermore, against U251 and U87 cell lines, alantolactone (**11**) reported IC_50_ values significantly lower than those of celecoxib (CB), a classical and potent commercial COX-2 inhibitor, which reported IC_50_ values of 120.32 μM and 135.27 μM, respectively [96]. In addition, this study [96] also found that the antitumor effect of alantolactone (**11**) in the GBM cells could be in part via NF-κB/COX-2-mediated signaling cascades through inhibition of IKKβ kinase activity. As referred above, the overexpression of COX-2 has been associated with inflammatory processes and also related with carcinogenesis and resistance to apoptosis [50,51]. Since IKKβ is the major subunit of this complex, its inhibition by alantolactone (**11**) ultimately leads to a decrease in the COX-2 expression and consequent intensification of the cytotoxic effect in the cells. Taking into account the results of the in vitro studies, the authors [96] also investigated the possible therapeutic effect of alantolactone (**11**) against tumor growth in BALB/c male nude mice. They noticed that toxic effects were not detected in the mice treated only with alantolactone (**11**) (10 and 20 mg/kg b.w.), and tumor weights and volumes decreased in the study group when compared with the control group (tumor inhibition rates of 47.73 ± 9.32% and 70.45 ± 13.33%, respectively).

Alantolactone (**11**) seems to be a very versatile compound. Not only due to its activities referred to in the previous points, but also because it exhibits cytotoxic activity against solid tumors, as referred to in the previous paragraph, and also against nonsolid tumors, as shown by Ding et al. [97]. In this work [97], alantolactone (**11**) shows selective (SI > 8) antitumor activity against several acute myeloid leukemia stem cell lines (AML), such as THP-1 (IC_50_ = 2.17 ± 0.72 μM), KG1a (IC_50_ = 2.75 ± 0.65 μM), K562 (IC_50_ = 2.75 ± 0.64 μM), and HL60 (IC_50_ = 3.26 ± 0.88 μM), as well as in the multidrug-resistant cell lines K562/A02 (IC_50_ = 2.73 ± 0.83 μM) and HL60/ADR (IC_50_ = 3.28 ± 0.80 μM), where alantolactone (**11**) is more cytotoxic than the clinically used drug adriamycin (ADR) (IC_50_ = 8.94 ± 3.79 μM against K562/A02 and IC_50_ = 5.54 ± 1.21 μM against HL60/ADR). Unfortunately, the results of this work should be considered under reserve, since the associated standard deviation is very high (about 20% of the mean). Above all, this applies to the cytotoxicity of the clinical drug against the K562/A02 multiresistant cell line, where the standard deviation reaches 42% of the mean value, which means a high dispersion of the results obtained in different replicates and, therefore, a low confidence in the result. The authors [97] also noticed that treatment with alantolactone (**11**) on HL60 and KG1a cell lines caused induction of cellular apoptosis by suppression of the NF-κB pathway, an important pathway involved in oxidative-stress related complications. An overexpression of the pro-apoptotic protein Bax was observed, while the expression of Bcl-2, an apoptosis inhibitor, and of NF-κB p65 subunit were reduced significantly. The alantolactone also caused the reduction of the downstream target proteins of the NF-κB pathway, the X-linked inhibitor of apoptosis protein (XIAP) and the FLICE-inhibitory protein (FLIP) that play important roles in cell apoptosis [97].

1-*O*-Acetylbritannilactone (**12**) (Figure 2), like alantolactone (**11**) (Figure 2), is a sesquiterpene lactone very common in *Inula* species [12] that elicits apoptosis in cancer cell lines through partially targeting the NF-κB pathway [98]. In fact, Wang et al. [98] showed that the combination of 1-*O*-acetylbritannilactone (**12**) (10 μM) and the approved chemotherapy drug gemcitabine (10 μg/mL) had a synergistic effect on the suppression of A549 cells proliferation, by inducing apoptosis in a 72 h treatment. The mixture decreases significantly the cell survival rates (mix of the two compounds cell survival = 30.2%) when compared with the control (100%), and with the compounds alone (1-*O*-acetylbritannilactone = 59.1%; gemcitabine alone = 49.7%). The authors also found that 1-*O*-acetylbritannilactone (**12**) and the combination treatment significantly decreased the expression of NF-κB and Bcl-2, while upregulating Bax expression [98].

Angiogenesis is a complex and normal process that allows the formation of new blood vessels (capillary formation) from the pre-existing ones, being crucial during wound healing or embryo development; however, it is abnormally present in cancer [99]. As a critical component of tumor angiogenesis, glycoprotein vascular endothelial growth factor (VEGF) is widely expressed in many cancers [100,101], while the vascular endothelial growth factors receptor-2 (VEGFR2) increased signaling is also characteristic of angiogenesis in tumors [102,103,104]. Alantolactone (**11**) (Figure 2) exhibits anti-angiogenesis property, since it shows anti-proliferative activity against human umbilical vascular endothelial cells (HUVEC) (IC_50_ = 14.2 μM), a model cell line used to study angiogenesis processes [105]. The alantolactone (**11**) anti-angiogenesis property could be related with its capacity to decrease capillary formation, by suppressing VEGFR2 signaling and decreasing the expression of its multiple downstream protein kinases, e.g., focal adhesion kinase (FAK) [105].

Anti-angiogenic activity is also exhibited by 1-*O*-acetylbritannilactone (**12**) (Figure 2) [106]. In the in vitro assay, 1-*O*-acetylbritannilactone (**12**) at 5 μM and 10 μM dose-dependently inhibits VEGF (25 ng/mL)-stimulated HUVEC migration, proliferation, and capillary structure formation [106]. Regarding the in vivo assay, administration for 20 consecutive days of 1-*O*-acetylbritannilactone (**12**) (12 mg/kg b.w. per day) to A549 tumor xenografts male nude BALB/c mice cause a significant decrease in tumor cell angiogenesis and tumor growth when compared to the control group, without significant toxicity or adverse effects to the experimental animals [106]. The 1-*O*-acetylbritannilactone (**12**) seems to have the ability to suppress the VEGFR2 downstream Src-FAK signaling pathway, by remarkable inhibition of steroid receptor coactivator (Src) and FAK phosphorylation [106]. This last two are crucial signaling kinases in VEGF-mediated angiogenesis, by working together, or separately, to promote growth, migration, and survival of endothelial cells as well as capillary tube formation [100,101].

**Figure 4:**
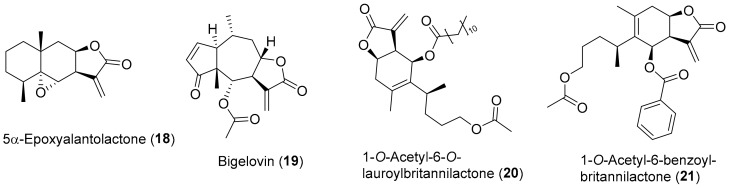
Chemical structure of *Inula* secondary metabolites (**18**–**19**) and semisynthetic derivatives (**20**–**21**) with reported activity against oxidative-stress carcinogenesis.

Another study [107] found that the 5α-epoxyalantolactone (**18**) (Figure 4), a sesquiterpene lactone isolated from the roots of *Inula helenium* and with a chemical structure very similar to alantolactone (**11**), had antiproliferative activity against human leukemia stem-like cell line KG1a. It presents an IC_50_ value of 3.36 ± 0.18 μM and was found to reduce the expression of anti-apoptotic protein Bcl-2 and increased the expression of pro-apoptotic protein Bax in a dose-dependent manner, while increasing the release of cytochrome into the cytoplasm, culminating in apoptosis of the cells [107].

Several important physiological functions in inflammation, cell differentiation, proliferation, and cell survival, as well as apoptosis and immune modulation are mediated by many cytokines [108,109]. The activation of the cytokines signals transduction of the Janus kinase (JAK) and STAT pathway, where JAKs phosphorylate STATs, causing their activation, associated with cancer and other proliferative diseases [110,111]. A study [112] showed that bigelovin (**19**) (Figure 4), a very abundant sesquiterpene lactone found in several *Inula* species [12,13], is a potent inhibitor of the JAK2/STAT3 signaling pathway. It directly inactivates JAK2 and blocks the downstream signaling transduction pathway, blocking IL-6-induced activation of STAT3. This explains the bigelovin (**19**) remarkable antitumor activity against several cancer cell lines from different tissues [112,113], e.g., human lung carcinoma cell lines (A549 IC_50_ ≅ 4.5 μM and H460 IC_50_ ≅ 8.5 μM), human cervical carcinoma cell line (HeLa IC_50_ ≅ 3.3 μM), human hepatocellular carcinoma cell line (HepG2 IC_50_ ≅ 7.1 μM), human breast adenocarcinoma cell line (MDA-MB-231 IC_50_ ≅ 1.3 μM, MDAMB-453 IC_50_ ≅ 2.5 μM and MDA-MB-468 IC_50_ ≅ 1.1 μM), and human leukemia cell lines (HL-60 IC_50_ ≅ 0.5 μM, Jurkat IC_50_ ≅ 0.9 μM and U937 IC_50_ ≅ 0.6 μM) [112]. Li et al. [114] showed that bigelovin (**19**) also acts mainly via the IL6/STAT3 pathway, significantly and effectively exerting anti-inflammatory and antitumor effects on colorectal cancer cells (CRC). In in vitro assay, cell viability, proliferation and colony formation of colon cancer cells colon-26 and its most aggressive version colon-26-M01 cells are inhibited in time- and dose-dependent manners, by bigelovin (**19**), with IC_50_ values of 0.99 ± 0.3 μM and 1.12 ± 0.33 μM, respectively [114]. In in vivo assay, the male BALB/c mice inoculated with human colon adenocarcinoma cell line HCT 116 and murine colon cancer cell line 26-M01 were subjected to treatment with bigelovin (**19**), at 0.3, 1, and 3 mg/kg b.w., applied every three days for 6 times. All doses significantly suppressed tumor growth and inhibited metastasis without decrease of body weight in both CRC mouse models [114].

As confirmed by all the above, several compounds isolated from *Inula* species exhibit relevant properties in the fight against oxidative-stress related diseases, with 1-*O*-acetylbritannilactone (**12**) (Figure 2) being one of the most studied compounds. The interest in this compound led to the publication of several studies on the synthesis of derivatives and evaluation of their biological activity. In some cases, the results obtained are very interesting. For example, the semisynthetic derivative 1-*O*-acetyl-6-*O*-lauroylbritannilactone (**20**) (Figure 4) is one of the most promising 1-*O*-acetyl-britannilactone derivatives (it bearing a lauroyl group at C-6 position) and exhibits cytotoxic activity against several cell lines (HCT 116, HEp-2 and HeLa), with IC_50_ values of 2.91 ± 0.61 μM, 5.85 ± 0.45 μM, and 6.78 ± 0.23 μM, respectively [115]. It is not so effective as etoposide (IC_50_ values of 2.13 ± 0.23 μM, 4.79 ± 0.54 μM, and 2.97 ± 0.25 μM, respectively) but a lot better than 1-*O*-acetylbritannilactone (**12**), (IC_50_ values of 36.1 ± 3.1 μM, 19.3 ± 1.5 μM, and 32.6 ± 2.5 μM, respectively) [115]. It should be noticed that, at least in the case of the HCT 116 cell line, 1-O-Acetyl-6-O-lauroylbritannilactone (**20**) could rival etoposide while being less toxic to the CHO normal cell line (IC_50_ = 5.97 ± 0.12 μM) than the reference compound etoposide (IC_50_ = 2.60 ± 0.15 μM). In addition, 1-O-Acetyl-6-O-lauroylbritannilactone (**20**) was also found to cause cell-cycle arrest in the G2/M phase in HCT 116 cell line [115].

In a similar work [116], the 6-OH position of 1-*O*-acetylbritannilactone (**12**) was modified with a variety of substituents, being the semisynthetic derivative, 1-*O*-acetyl-6-benzoyl-britannilactone (2**1**) (Figure 4), the most promising antitumor derivative with IC_50_ values of 5.19 ± 0.10 μM and 9.93 ± 0.06 μM against HeLa and SGC-7901 cell lines, respectively, an activity level not much different from those of reference drug etoposide (HeLa IC_50_ = 2.97 ± 0.25 μM and SGC-7901 IC_50_ = 6.56 ± 0.68 μM), but it does not rival with a 5-fluorouracil drug against SGC-7901 cell line (IC_50_ = 0.86 ± 0.05 μM) [116]. In addition to this, it is worth mentioning that this type of approach is very interesting and worth investing in, because the adequate structural modification of the natural compounds enables the development of new affordable, efficient, and safe antineoplastic drugs [117].

As referred above, under impaired antioxidant pathways, critical cellular gene mutations can be induced by oxidative stress, which can be the major carcinogenic inductor [7,118]. However, in some cases, the increase in oxidative stress levels could also contribute to antitumor activity [119]. In fact, alantolactone (**11**) (Figure 2) [120] and bigelovin (**19**) (Figure 4) [121], two compounds described above as cytotoxic agents by antioxidant pathways, can have cytotoxic activity also through pro-oxidant pathways. These two studies [120,121], among several in the literature [119,121,122,123], are presented here as examples of a new perspective on the role of ROS, showing that in some cases, the production of ROS may be beneficial. In fact, the cytotoxic activity by pro-oxidant action opens new perspectives in research on the role of ROS species in biological systems as well as on new ways of fighting cancer. However, understanding the factors related to the cytotoxic effect by pro-oxidant mechanism and its effects in an integrated perspective require much more in-depth studies. Its discussion in more detail, although interesting, falls outside the scope of this review.

## 4. Conclusions

Taking into account the recent literature presented on this review regarding compounds with antioxidant properties and action mechanisms that target the reduction of the oxidative stress nature inherent to the various mentioned diseases, it should be mentioned that many aspects still require clarification and further studies. Knowledge about the interactions of the mentioned compounds with others, as well as the precise pathways through which some compounds exert their therapeutic activities remains scarce. The *Inula* species showed to be a good source of interesting and active compounds that act against oxidative-stress related diseases, through antioxidant mechanisms and/or other nonspecific antioxidant pathways, culminating in a melioration of the oxidative-stress induced problems. From all compounds, β-caryophyllene (**2**) is one of the most promising ones, since it presented higher antioxidant activity in the DPPH assay (IC_50_ of 1.25 ± 0.06 μM), more active than the reference ascorbic acid. Jaceoside (**14**) exhibits the best anti-inflammatory activity from all compounds (IC_50_ of 0.34 ± 0.01 μM), through inhibition of NO production. Jaceoside (**14**) should be taken in consideration as another promising compound for future studies regarding different bioactivities and its mechanisms of action. Alantolactone (**11**) is the most polyvalent compound, reporting interesting IC_50_ values for several bioactivities (i.e., anti-inflammatory, anti-diabetic, neuroprotective, and antitumoral). 1-*O*-acetylbritannilactone (**12**) can be also pointed out as a promising compound, since it can be used as a blueprint for the development of analogues with interesting properties. This work expects to highlight the relevance of *Inula* species as a source of compounds with relevant bioactivities against stress-oxidative related diseases.

## Figures and Tables

**Table 1:** Scavenging effects of *Inula* secondary metabolites **1**–**10** and reference compound on 1,1-diphenyl-2-picrylhydrazyl (DPPH) and 2,2’-azino-bis(3-ethylbenzothiazoline-6-sulphonic acid) (ABTS) radicals (EC_50_, μM).

Compound	DPPH (Reference Compound)	ABTS (Reference Compound)	*Inula* Source
1,3-dicaffeoylquinic acid (**1**)		12 ± 0.4 (Ascorbic acid: 15 ± 0.01) [25]	*Inula helenium* [26]
β-caryophyllene (**2**)	1.25 ± 0.06 (Ascorbic acid: 1.5 ± 0.03) [27]		*Inula cappa* (Buch.-Ham. ex D.Don) DC. * [28]
Caffeic acid (**3**)	25.0 ± 1.7 (Ascorbic acid: 20.7 ± 1.31) ** [29]	8.82 ± 0.33 (Ascorbic acid: 15.05 ± 2.61) ** [29]	*Inula helenium* [30]
Chlorogenic acid (**4**)	36.83 ± 0.76 (Caffeic acid: 35.02 ± 2.11) ** [31]		*Inula ensifolia* L. [32], *Inula cappa* [33], *Inula helenium* [34]
Isoquercitrin (**5**)	12.68 ± 0.54 (Trolox: 18.10 ± 0.44) ** [35]		*Inula japonica* [36], *Inula ensifolia* [32], *Inula helenium* [34]
Kaempferol (**6**)	27.18 ± 1.05 (Ascorbic acid: 20.72 ± 1.31) ** [29] 47.97 ± 0.03 (Ascorbic acid: 20.27 ± 0.11) ** [37]	12.93 ± 0.52 (Ascorbic acid: 15.05 ± 2.61) ** [29]	*Inula salsoloides* (Turcz.) Ostenf. [38]
Luteolin (**7**)	6.69 ± 0.15 (Ascorbic acid: 16.88 ± 0.02) [39]		*Inula japonica* [36], *Inula salsoloides* [38], *Inula britannica* L. [40]
Quercetin (**8**)	8.80 ± 0.79 (Ascorbic acid: 20.72 ± 1.31) ** [29] 19.75 ± 1.06 (Caffeic acid: 35.02 ± 2.11) ** [31]	6.25 ± 1.09 (Ascorbic acid: 15.05 ± 2.61) ** [29]*	*Inula japonica* [36], *Inula britannica* [41], *Inula helenium* [34]
Quercitrin (**9**)	9.93 ± 0.38 (Trolox: 18.10 ± 0.44) [35]		*Inula japonica* [36], *Inula ensifolia* [32], *Inula helenium* [34]
Rutin (**10**)	19.31 ± 0.39 (Caffeic acid: 35.02 ± 2.11) ** [31]		*Inula helenium* [34]

* According to “The plant list” database [14], this is an unresolved name. ** After unit conversion from μg/mL to μM.

## Abbreviations

3T3-L1	Mouse adipocytes cells
26-M01	Murine aggressive colorectal cancer
A549	Human lung carcinoma
ABTS	2,2’-azino-bis(3-ethylbenzothiazoline-6-sulphonic acid
AChE	Acetylcholinesterase
AD	Alzheimer disease
ADR	Adriamycin
AML	Acute myeloid leukemia
Aβ	Amyloid-β
BALB/c	Strain of laboratory mouse
Bax	Bcl-2-associated X
Bcl-2	B-cell lymphoma 2
BV-2	Mouse microglia cells
b.w.	Body weight
C57BL/6J	Strain of laboratory mouse
CB	Celecoxib
CHO	Normal hamster cell line
CNS	Central nervous system
COX-2	Cyclooxygenase 2
CRC	Colorectal cancer
DNA	Deoxyribonucleic acid
DPPH	1,1-Diphenyl-2-picrylhydrazyl
DPP-IV	Dipeptidyl peptidase 4
FAK	Focal adhesion kinase
FLIP	FLICE-inhibitory protein
GBM	Glioblastoma multiforme
GLP-1	Glucagon-like peptide 1
GSH	Glutathione
GSR	Glutathione reductase
GSSG	Glutathione disulphide
H460	Human lung carcinoma
H9c2	Rat cardiomyoblasts
HaCaT	Nontumorigenic human epidermal cells
HAT	Hydrogen-atom transfer
HCT 116	Human colon cancer
HeLa	Human cervical carcinoma
HEp-2	Human larynx epidermal carcinoma
HepG2	Human hepatocellular carcinoma
HL-60	Human acute promyelocytic leukemia
HO-1	Heme oxygenase-1
HUVEC	Human umbilical vascular endothelial cells
IC_50_	Half maximal inhibitory concentration
IKK	IκB kinase
IκB-α	Inhibitory κB-α
IL-1	Interleukin 1
IL-1β	Interleukin-1β
IL-1R	Interleukin-1 receptor
IL-4	Interleukin 4
IL-6	Interleukin 6
IL-6R	Interleukin 6 receptor
iNOS	Inducible nitric oxide synthase
JAK	Janus kinase
Jurkat	Human acute T cell leukemia
K562	Human bone marrow chronic myelogenous leukemia
K562/A02	Human chronic myelogenous leukemia multidrug-resistant
KG1a	Human acute monocytic leukemia
LPS	Lipopolysaccharide
MAPK	Mitogen-activated protein kinase
MCP1	Monocyte chemoattractant protein 1
MDA	Malondialdehyde
MDA-MB-231	Human breast adenocarcinoma
MDA-MB-453	Human breast metastatic carcinoma
MDA-MB-468	Human breast adenocarcinoma (ethnicity: black)
MMP	Mitochondrial membrane potential
mRNA	Messenger ribonucleic acid
NCI-H716	Human colorectal adenocarcinoma
NF-κB	Nuclear factor kappa-B
NO	Nitric oxide
NQO1	NAD(P)H:quinone oxidoreductase-1
Nrf2	Nuclear factor erythroid 2-related factor 2
PANC-1	Human pancreatic epithelioid carcinoma
PGE2	Prostaglandin E2
PPARα	Peroxisome proliferation-activated receptor α
RAW 264.7	Macrophage normal cell line
RNA	Ribonucleic acid
ROS	Reactive oxygen species
RNS	Reactive nitrogen species
SET	Single-electron transfer
SGC-7901	Gastric carcinoma
SH-SY5Y	Human neuroblastoma
SI	Selectivity index
SMT	2-methyl-2-thiopseudourea sulphate
SOD	Superoxide dismutase
Src	Steroid receptor coactivator
STAT	Signal transducer and activator of transcription
STAT3	Signal transducer and activator of transcription 3
SVG	Normal human glial cell
TBI	Traumatic brain injury
THP-1	Human acute monocytic leukemia
TLR	Toll-like receptor
TNF-α	Tumor necrosis factor α
TNFR	Tumor necrosis factor receptor
TRAIL	TNF-related apoptosis inducing ligand
U87	Human primary glioblastoma
U118	Human glioblastoma
U251	Human glioblastoma
U937	Human histiocytic lymohoma
VEGF	Vascular endothelial growth factor
VEGFR2	Vascular endothelial growth factors receptor-2
XIAP	X-linked inhibitor of apoptosis protein
